# Supplementary material for: In situ observation of thermal-driven degradation and safety concerns of lithiated graphite anode
Source: Nat Commun. 2021 Jul 9;12:4235. doi: 10.1038/s41467-021-24404-1 (PMC8270978; doi:10.1038/s41467-021-24404-1)
Supplement: Supplementary file 1 — Supplementary Information [file 41467_2021_24404_MOESM1_ESM.pdf]

Supplementary Information

In situ observation of thermal-driven degradation and safety concerns of lithiated graphite anode

Xiang Liu,[1] Liang Yin,[2] Dongsheng Ren,[3,4] Li Wang,[3,4] Yang Ren,[2] Wenqian Xu,[2] Saul Lapidus,[2] Hewu Wang,[4] Xiangming He,[3,4] Zonghai Chen,[1] Gui-Liang Xu,[1]* Minggao Ouyang,[4]* and Khalil Amine[1,5,6]*

[1] Chemical Sciences and Engineering Division, Argonne National Laboratory, 9700 S. Cass Avenue, Lemont, IL 60439, USA

[2] X-ray Science Division, Argonne National Laboratory, 9700 S. Cass Avenue, Lemont, IL 60439, USA

[3] Institute of Nuclear and New Energy Technology, Tsinghua University, Beijing 100084, China

[4] State Key Laboratory of Automotive Safety and Energy, Tsinghua University, Beijing 100084, China

[5] Materials Science and Engineering, Stanford University, Stanford, CA 94305, USA

[6] Institute for Research& Medical Consultations (IRMC), Imam Abdulrahman Bin Faisal University (IAU), Dammam, Saudi Arabia

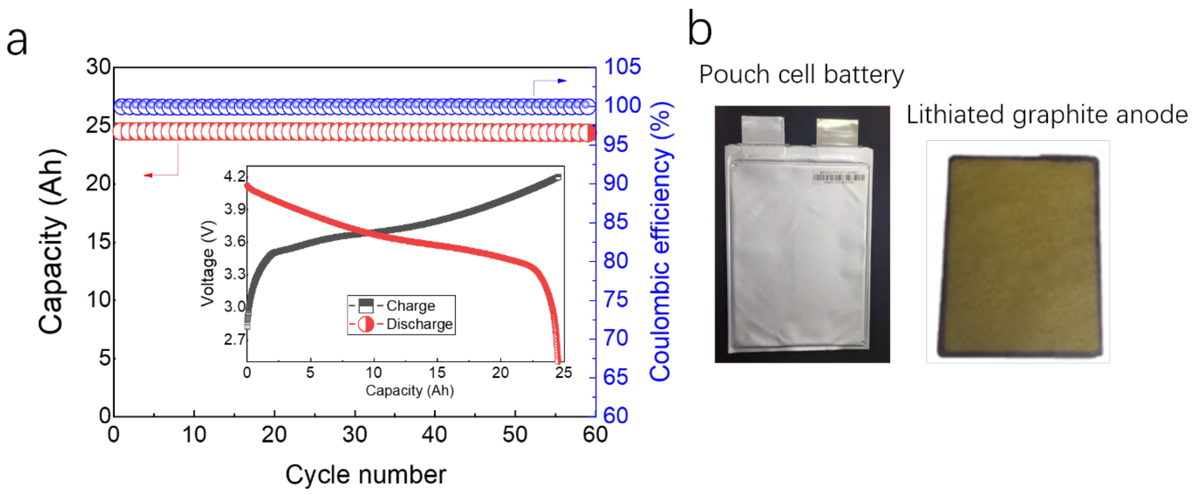

C Battery Specifications

Item	Specification
Cathode	$LiNi_{1/3}Mn_{1/3}Co_{1/3}O_2$
Anode	Graphite (95 wt.%) PVDF (2 wt.%) Carbon black (3 wt.%)
Electrolyte	1.2 M $LiPF_6$ in ethylene carbonate(EC): ethyl methyl carbonate(EMC)=3:7 (volume ratio)
Nominal capacity	24 Ah
Charging cut-off voltage	4.2 V
Discharging cut-off voltage	2.5V
size	227*160*7 mm

Supplementary Figure 1. The commercial 24 Ah electric vehicle-scale lithium-ion battery pouch cell. (a) The cycling performance of the pouch cell, cycled with 1C at 25°C; inset figure contains the charge/discharge curves between 2.5 V and 4.2 V, 1C at 25°C. (b) The digital photos of the lithium-ion battery pouch cell and the harvested lithiated graphite anode after being charged to 4.2 V (the golden color of LiC_6). (c) Battery specifications.

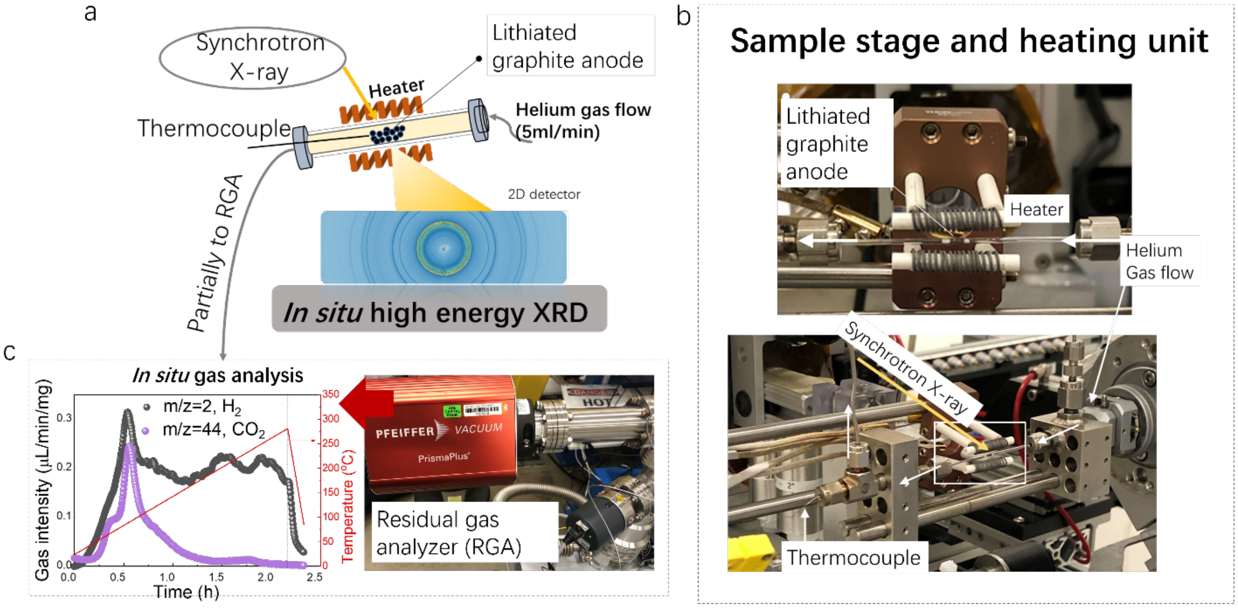

Supplementary Figure 2. The setup for *in situ* synchrotron HEXRD during heating with residual gas analyzer (mass spectroscopy). (a) Schematic illustration of the *in situ* HEXRD and mass spectroscopy during heating. (b) The sample stage, flow gas channel, and heating unit of the *in situ* experimental setup. As shown, the lithiated graphite anode was loaded in the quartz capillary (7 mm in diameter) between two heaters with a controllable temperature sensor in the quartz tube, then the tube was sealed with helium protective gas during the heating experiment. (c) The residual gas analyzer (Pfeiffer QMG 220) and the obtained time-resolved mass spectrum representative of hydrogen (m/z=2) and carbon dioxide (m/z=44).

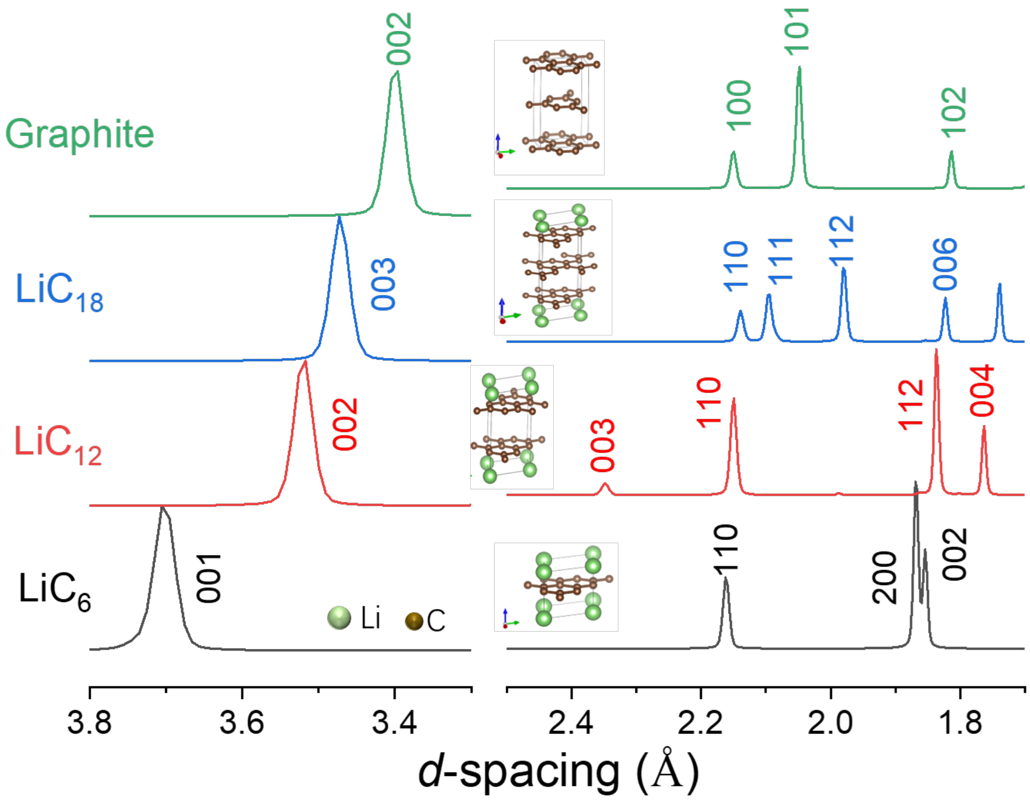

Supplementary Figure 3. Simulated standard Bragg reflections of graphite, LiC_{18}, LiC_{12}, and LiC_6. Inset are the lattice structures of graphite, LiC_{18}, LiC_{12}, and LiC_6, respectively.

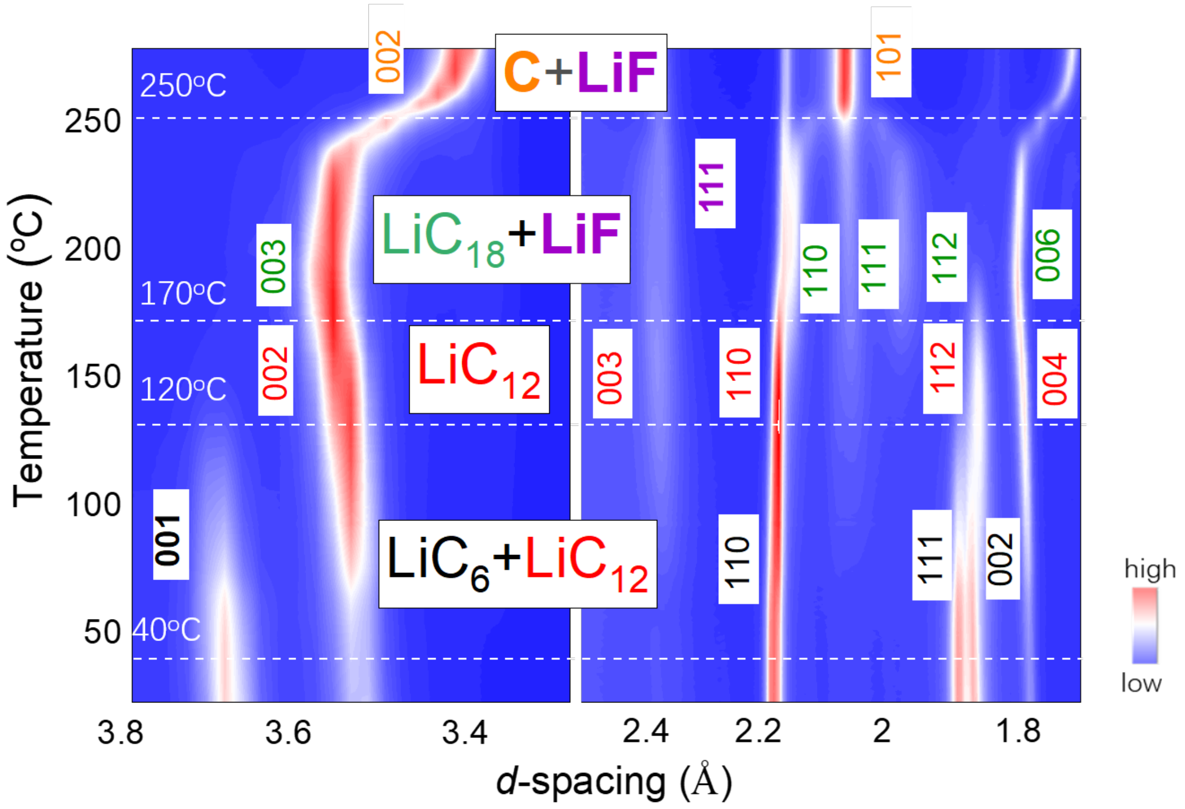

Supplementary Figure 4. The contour plots of lithiated graphite anode during heating from 25°C to 280°C with 2°C/min. The 2θ value has been converted into *d*-spacing following Bragg's rule for a better comparison to the results using different X-ray sources.

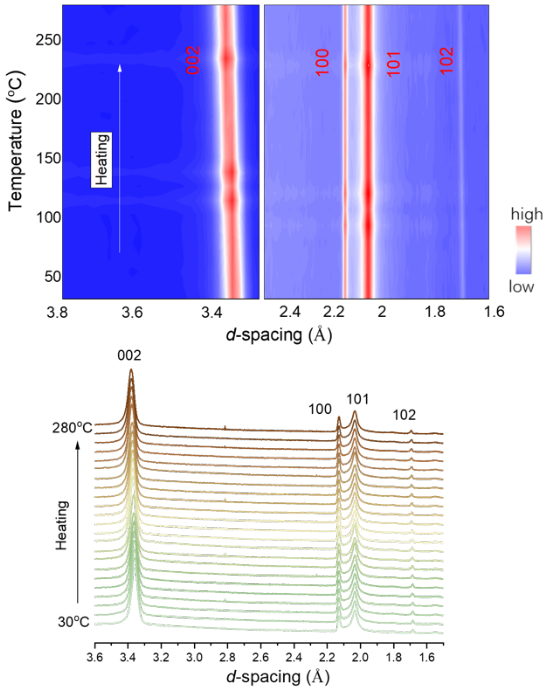

Supplementary Figure 5. The structural evolution of the unlithiated (uncharged) graphite anode with the same content of PVDF binder and conductive additives (carbon black) (95:2:3) during heating. (a) The contour plots and (b) waterfall plots of unlithiated graphite anode during the heating from 30°C to ~280°C at 2°C/min.

As shown, without lithium intercalation, the graphite showed no phase transformation when heated to 280°C. The graphite 2H (002), (100), (101), and (102) Bragg reflection only showed slight shifts to lower angles due to thermally induced lattice expansion. However, no phase transformation can be observed. The results confirmed that the unlithiated graphite is stable against the PVDF and carbon additive even at elevated temperature. However, with lithium intercalation in the graphite anode, the intercalated lithium is the reason for the instability of the lithiated graphite anode, which was gradually leached out of the graphite layer during the heating process.

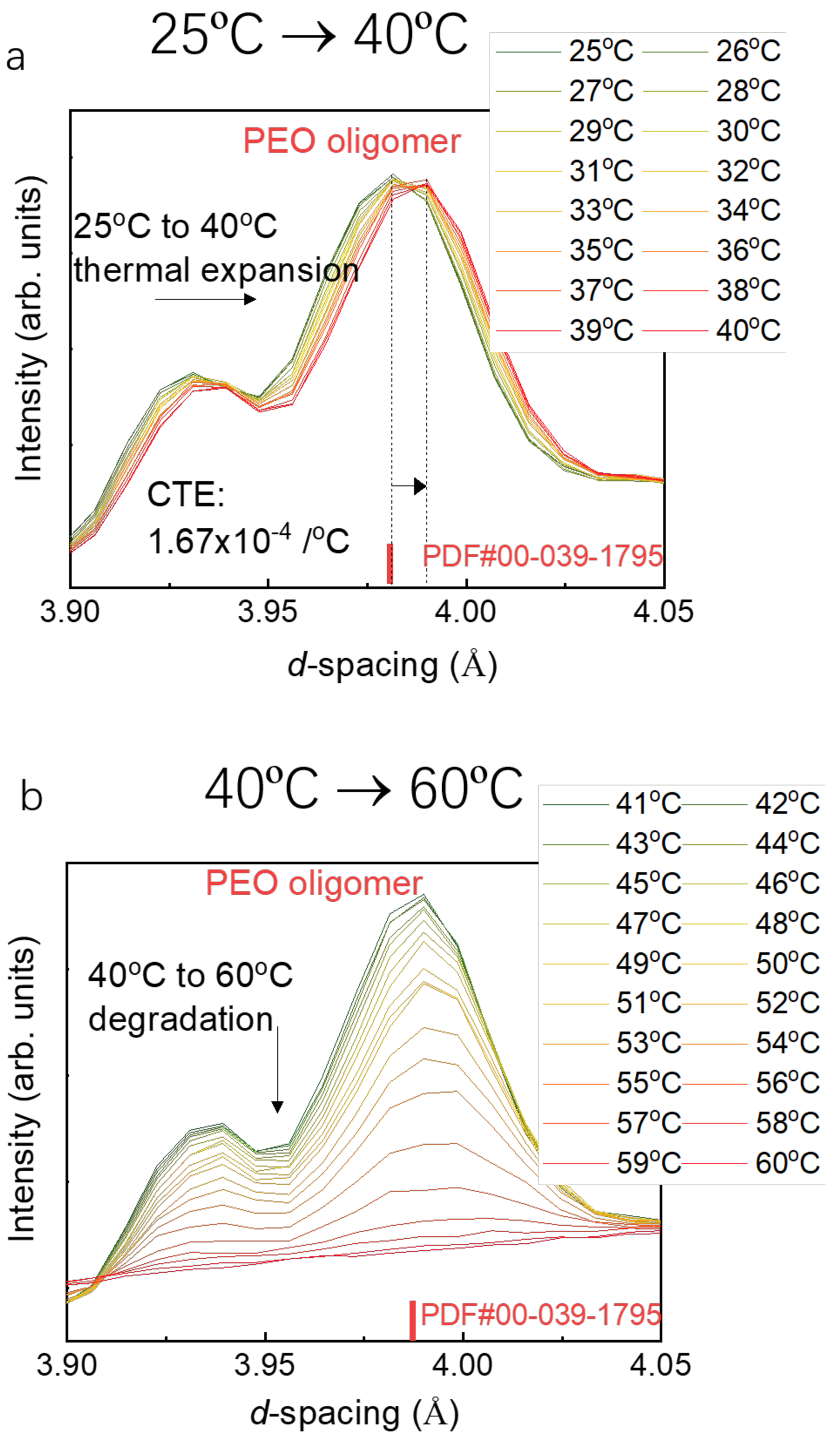

Supplementary Figure 6. (a) The PEO oligomer lattice expansion between 25°C~40°C, with a coefficient of thermal expansion (CTE) of 167×10-6/°C, (b) PEO degradation during 40°C~60°C.

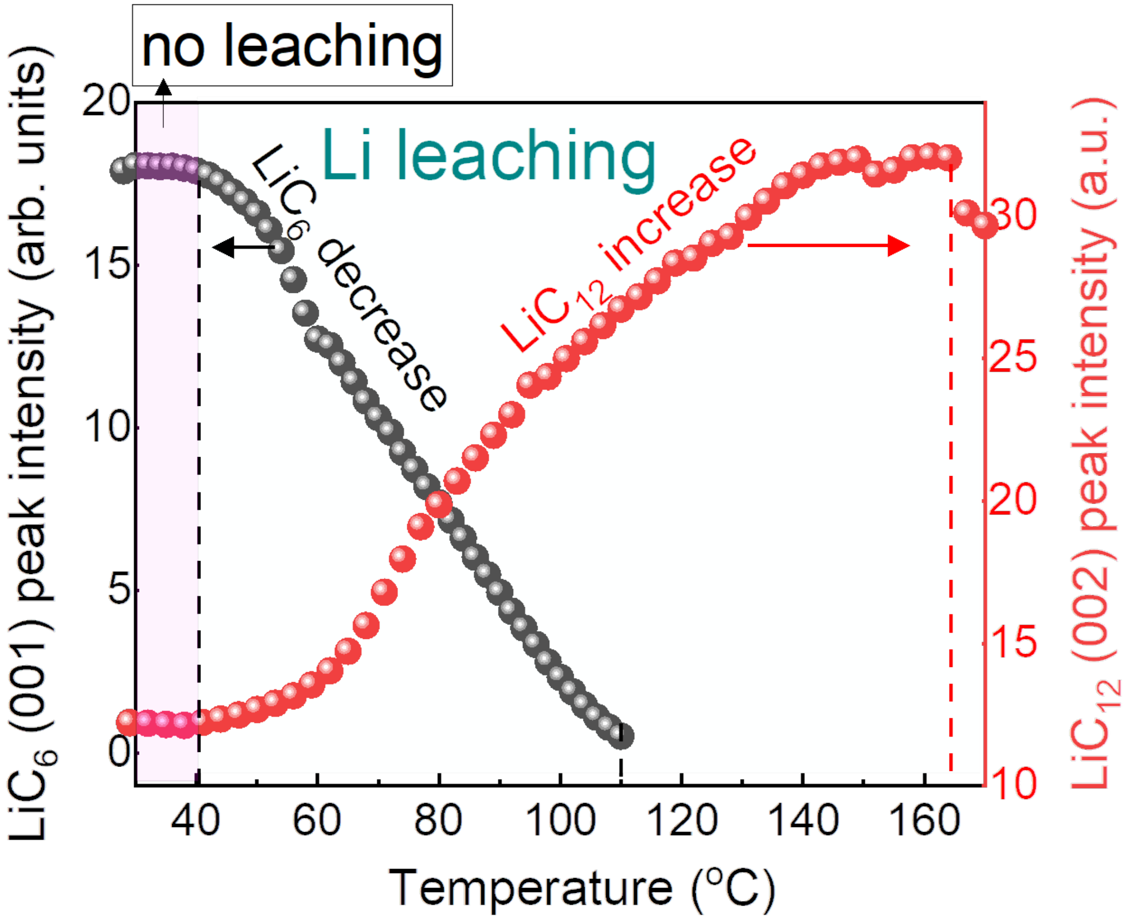

Supplementary Figure 7. The intensity evolution of LiC_6 (001) and LiC_{12} (002) Bragg reflections between 25°C and ~170°C.

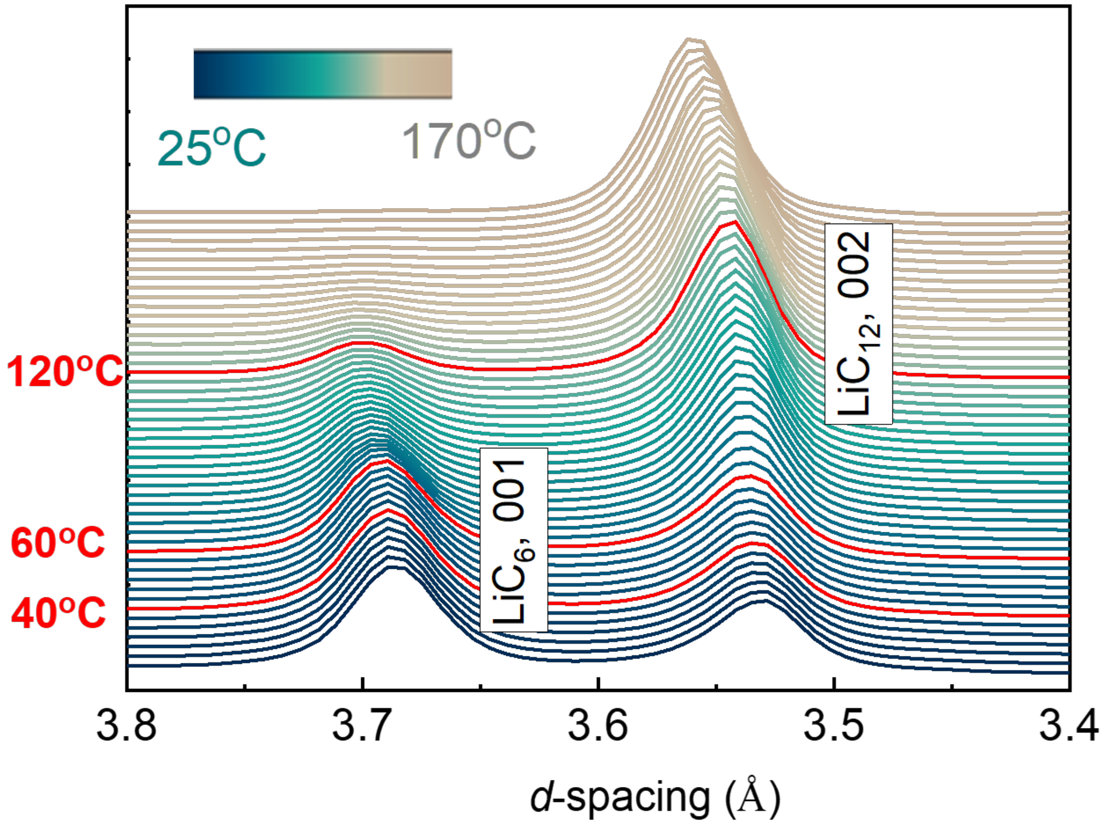

Supplementary Figure 8. The original HEXRD plots focusing on the LiC_6 (001) and LiC_{12} (002) Bragg reflections during the heating from 25°C to 170°C.

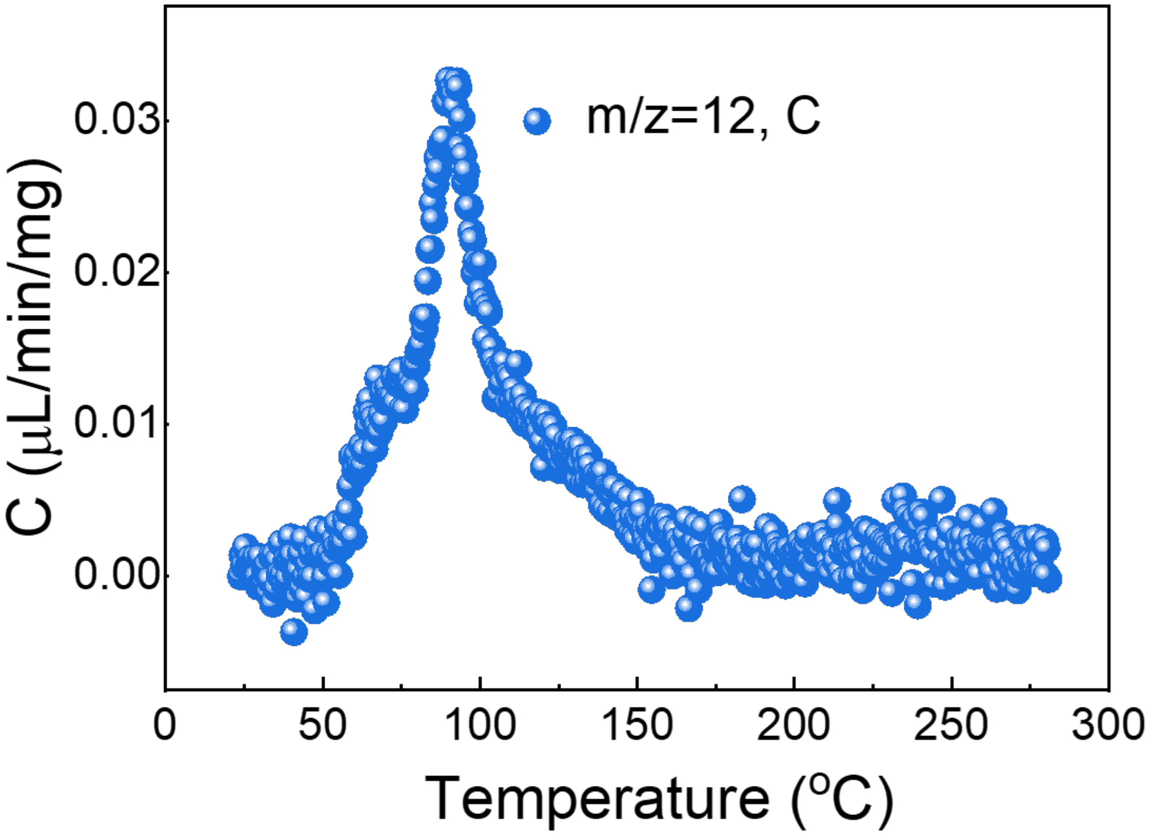

Supplementary Figure 9. The quantitative gas analysis of the carbon (m/z=12) during the heating process of the lithiated graphite anode.

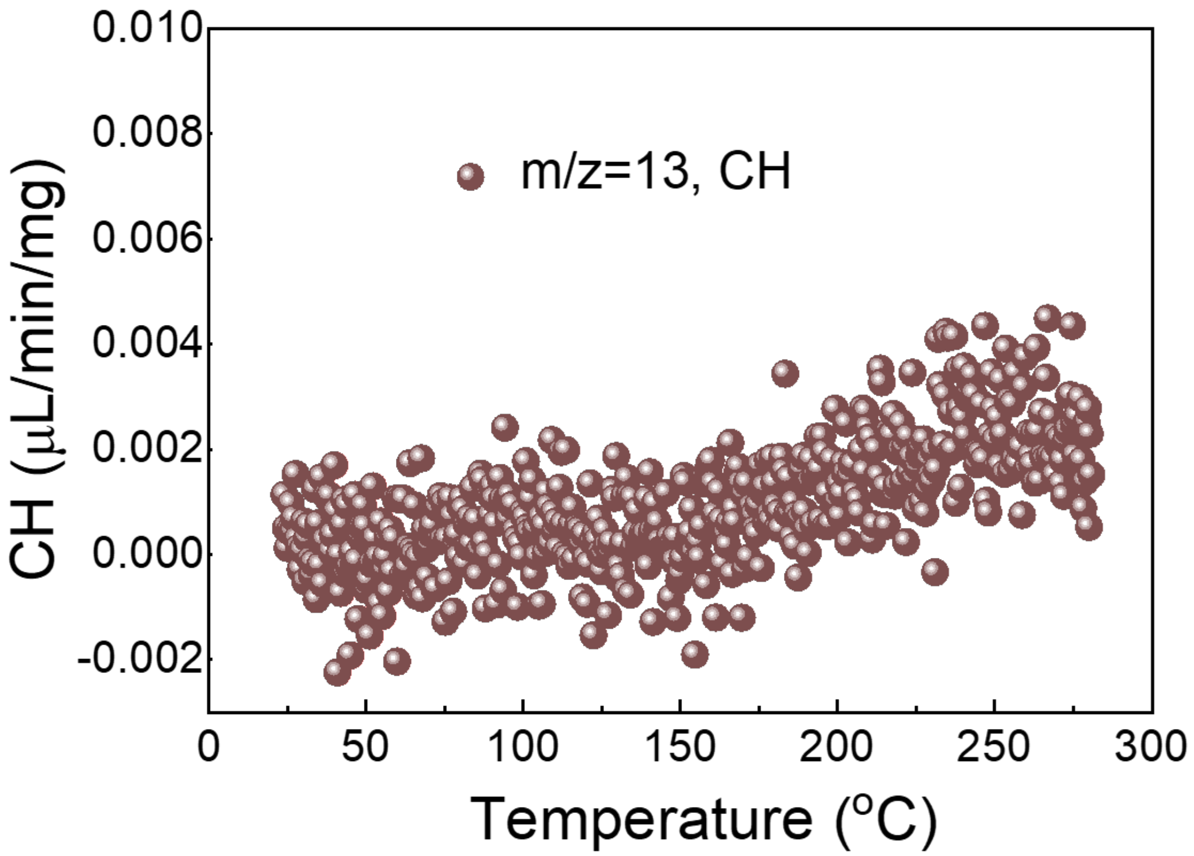

Supplementary Figure 10. The quantitative gas analysis of the CH fragment (m/z=13) during the heating process of the lithiated graphite anode.

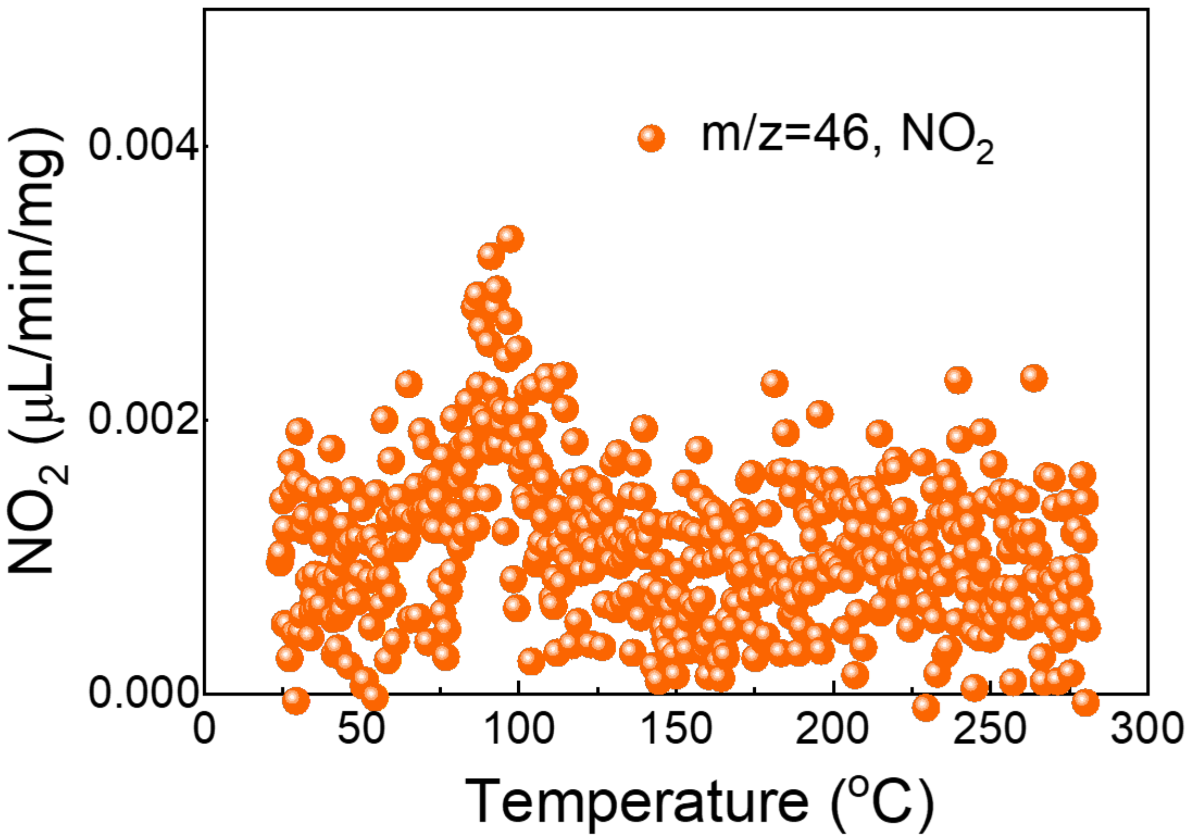

Supplementary Figure 11. The quantitative gas analysis of the NO_2 (m/z=46) during the heating process of the lithiated graphite anode.

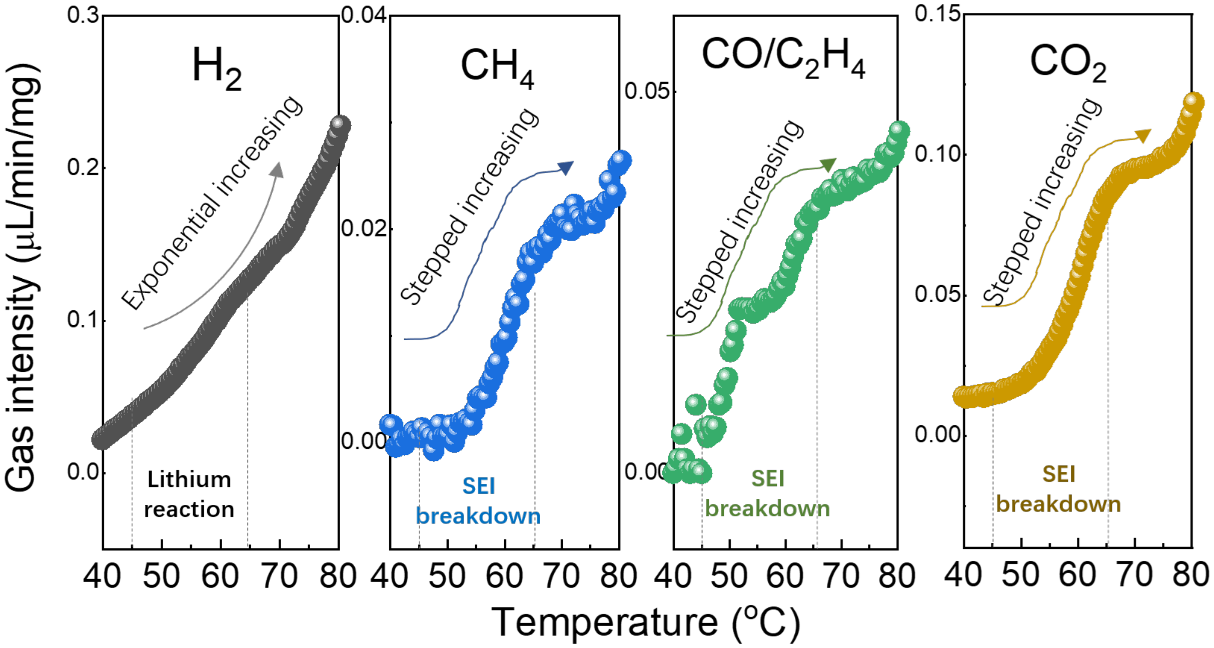

Supplementary Figure 12. Enlarged gas signals focusing on 40°C to 80°C for H_2, CH_4, CO/C_2H_4, and CO_2.

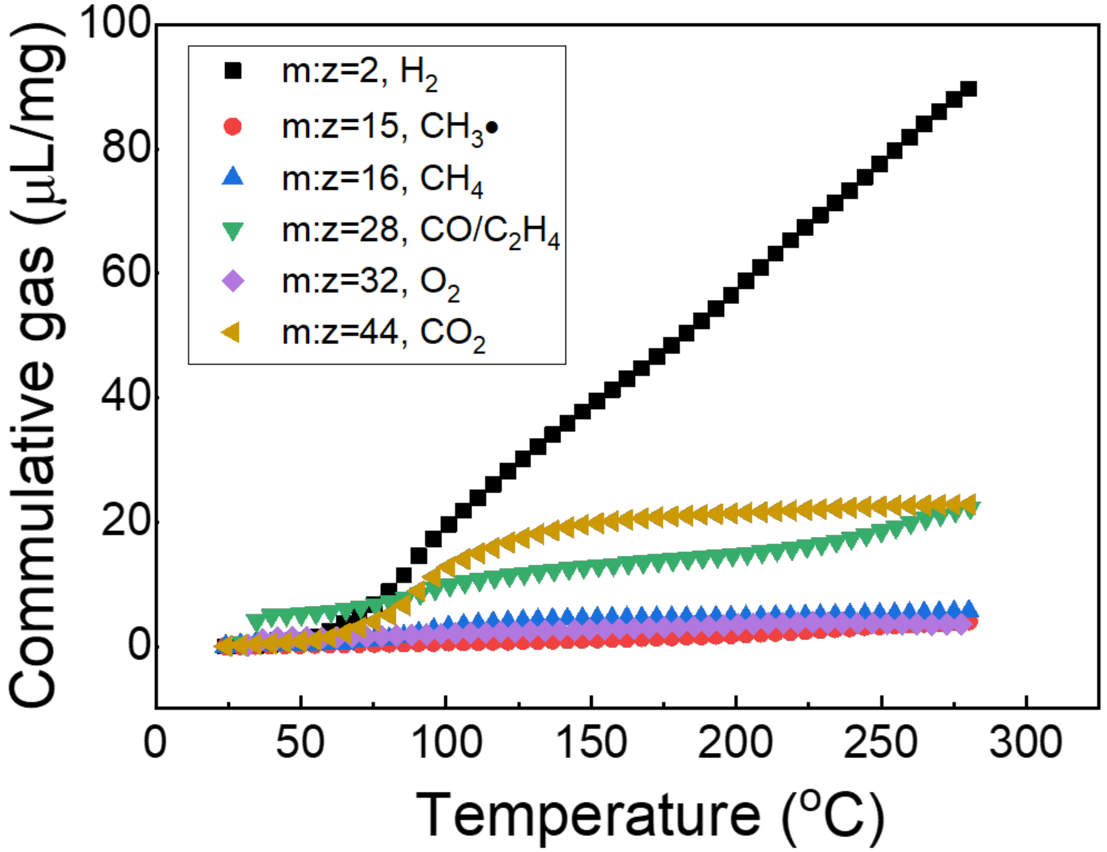

Supplementary Figure 13. A cumulative gas intensity of H_2, CH_3•, CH_4, CO/C_2H_4, O_2, and CO_2 gas during the heating from 25°C to 280°C.

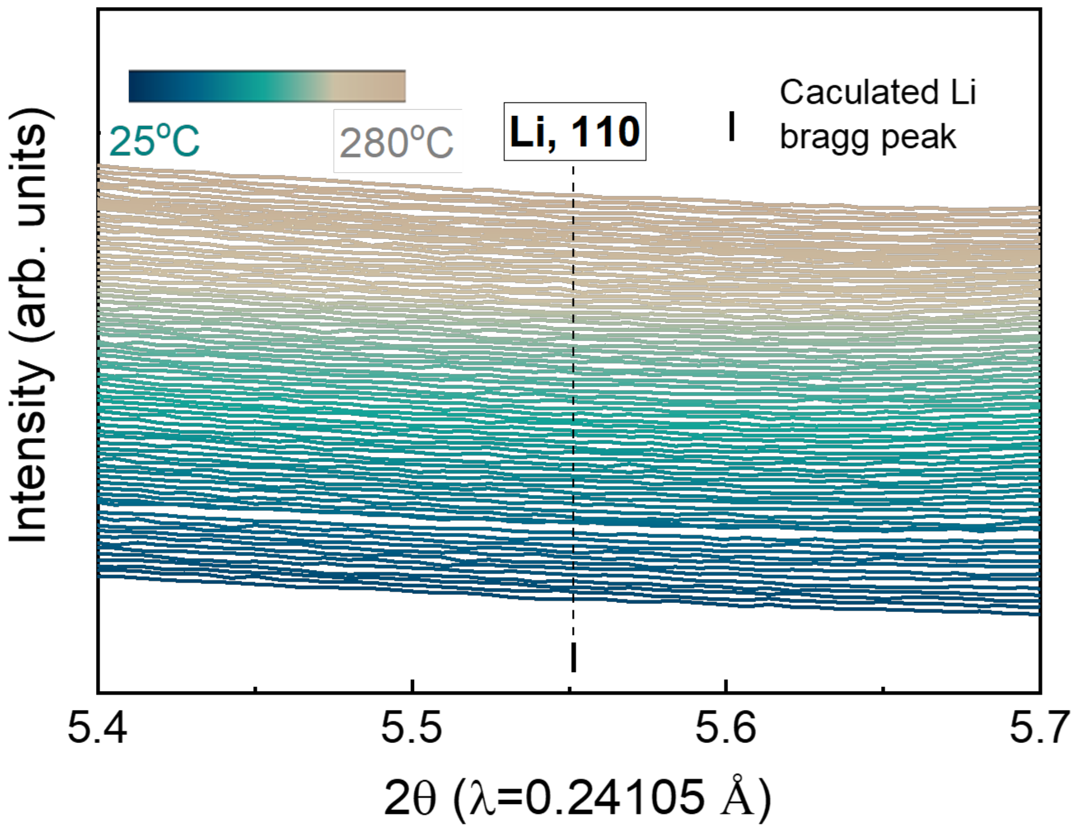

Supplementary Figure 14. *Operando* HEXRD plots of the lithiated graphite anode focusing on the lithium (110) Bragg reflection of 5.56° during the heating from 25°C to 280°C, indicating there is no ordered lithium *bbc* structure formation.

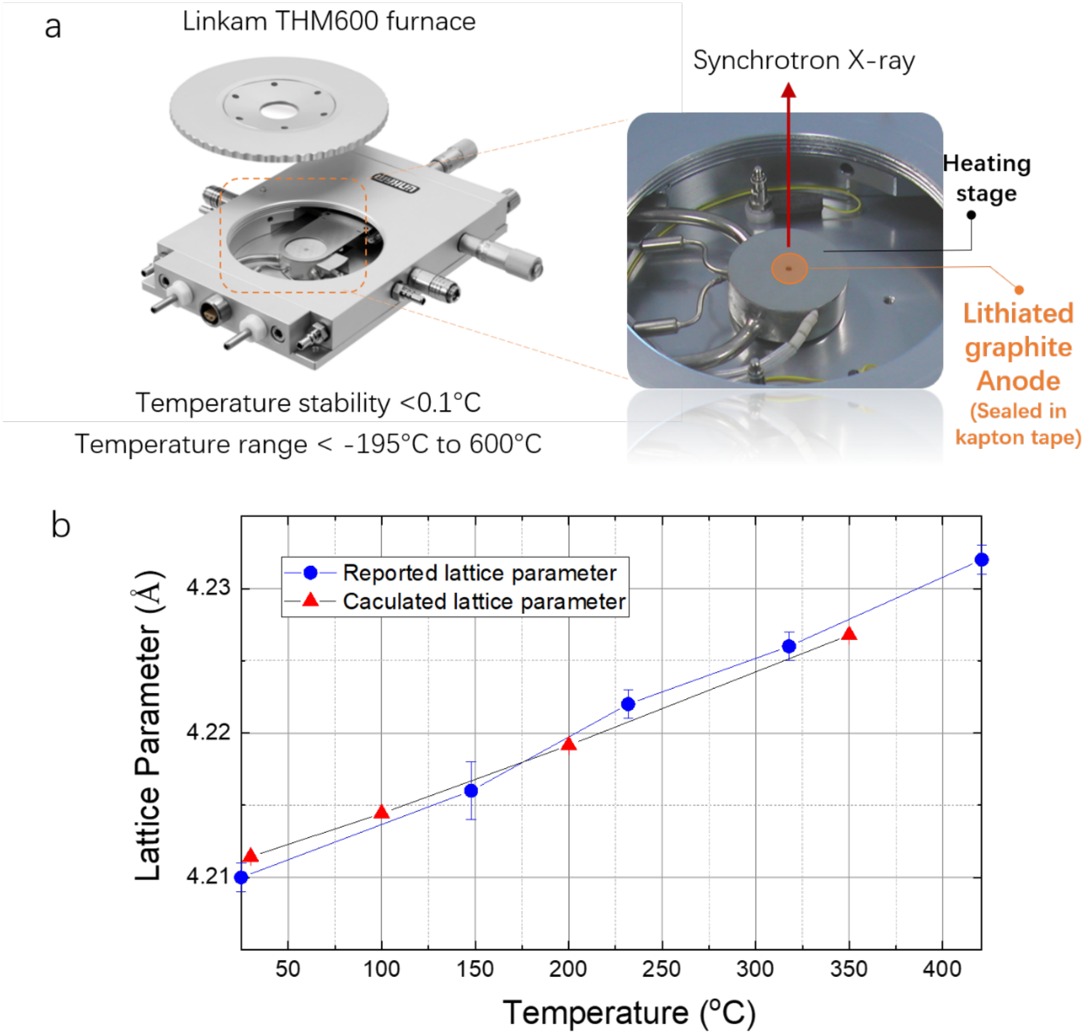

Supplementary Figure 15. (a)The experimental setup for the *in situ* PDF measurement of the lithiated graphite anode. (b) The temperature calibration data for the Linkam furnace by using a MgO as reference at the temperature range between 30ºC to 350ºC.

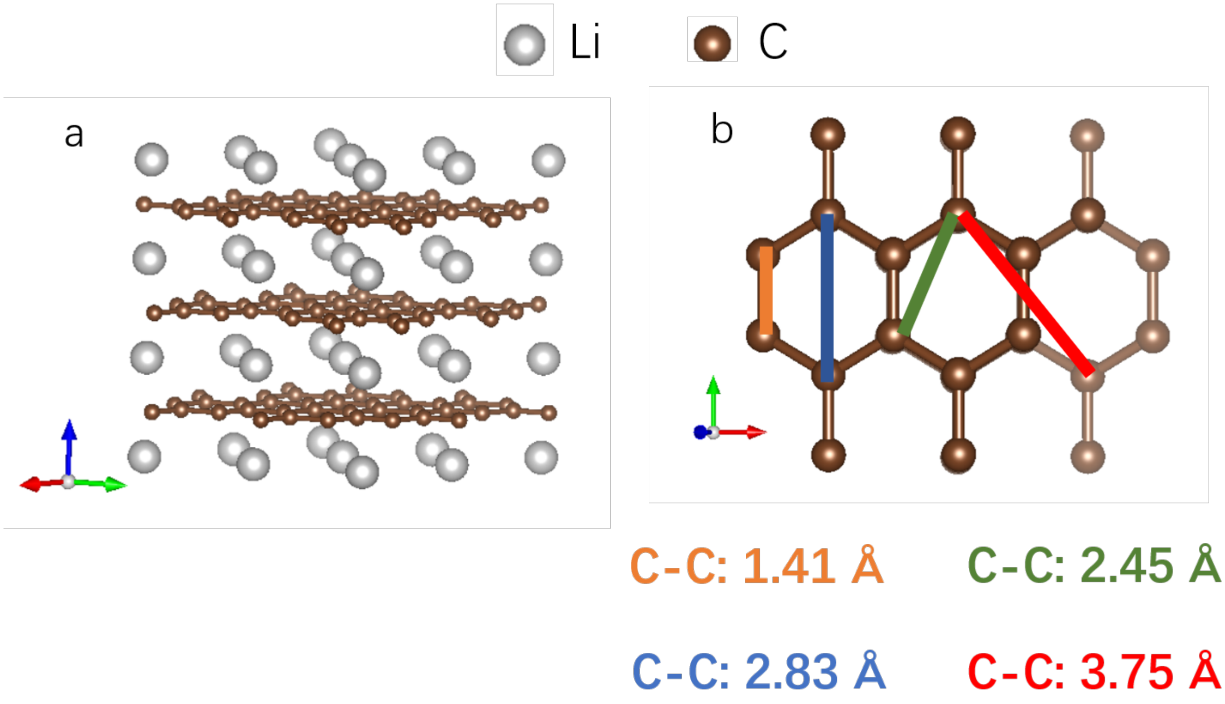

Supplementary Figure 16. (a) Illustration of the atomic structure of the lithiated graphite. (b) Illustration of the graphite showing different in-plane C-C bond distances.

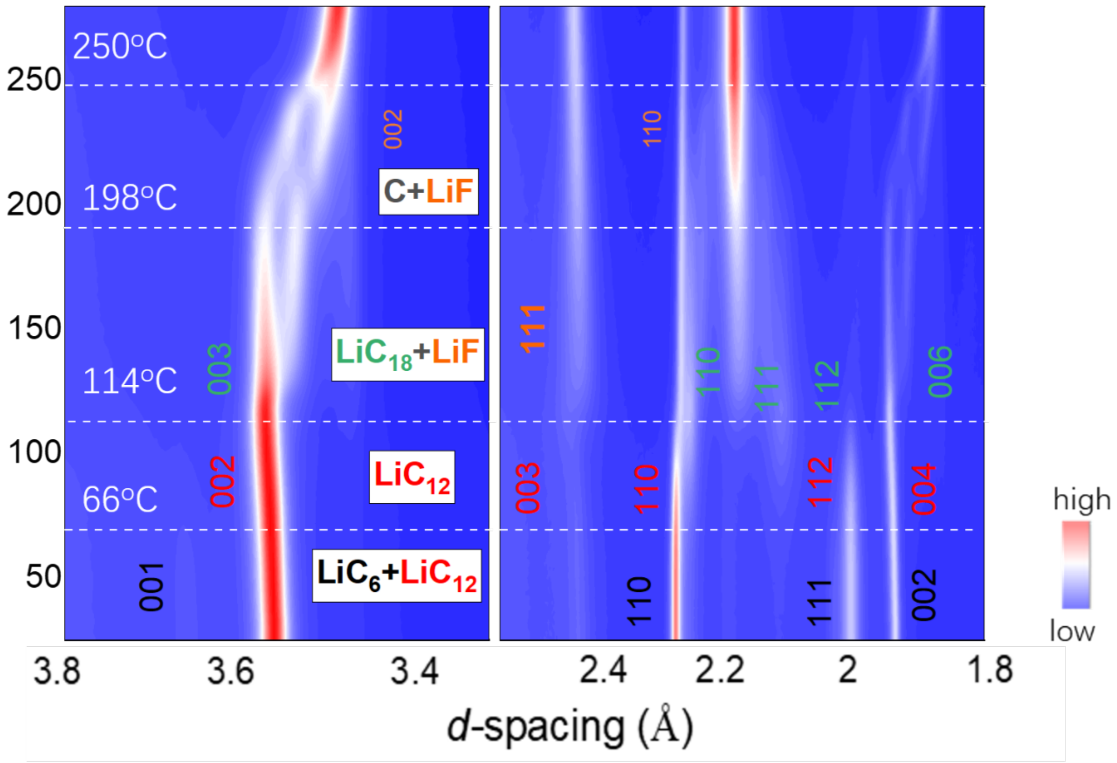

Supplementary Figure 17. The contour plot of the structural evolution of lithiated graphite with the presence of electrolyte during heating.

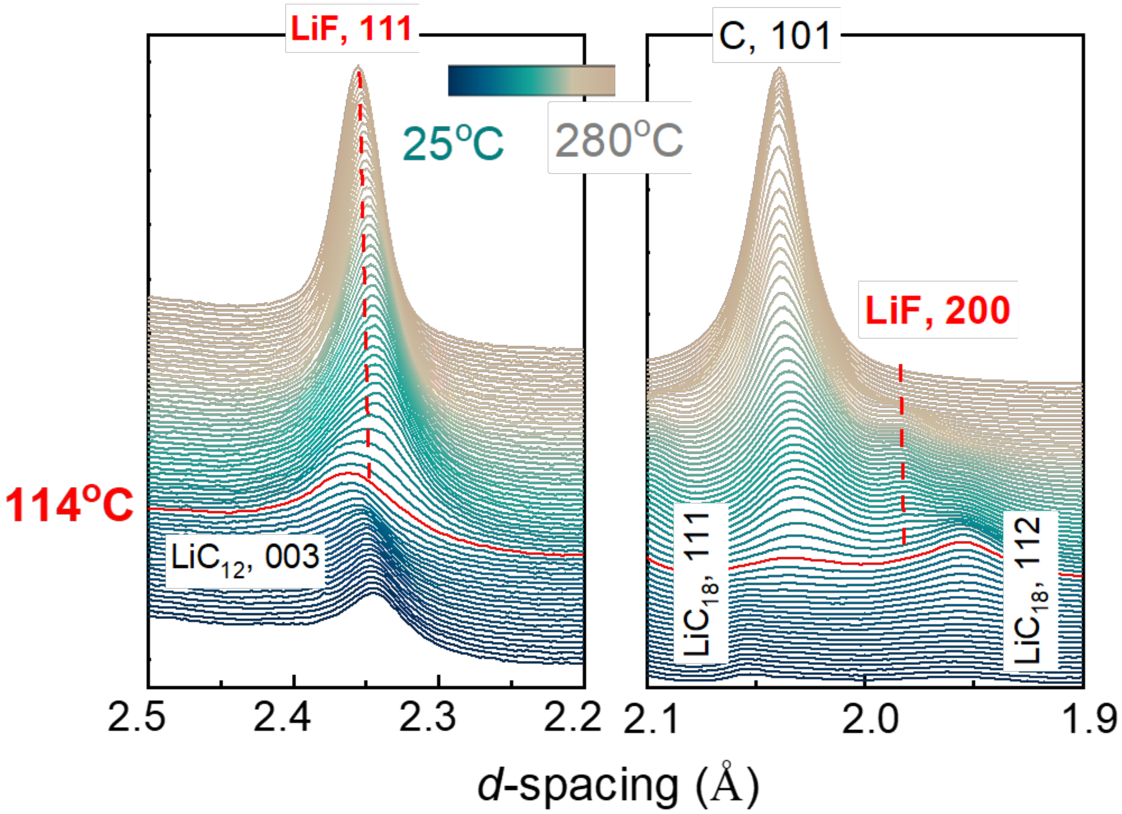

Supplementary Figure 18. The formation of LiF by tracing the LiF (111) and (200) Bragg reflections during the degradation of the lithiated graphite anode with the presence of electrolyte.

Supplementary Table 1. The weight ratio of leached lithium from the graphite anode based on HEXRD results

	Anode phase ratio		Anode elemental weight ratio		Leached lithium weight ratio
			Li	C	
**25°C**	LiC_6, 49 wt.%	LiC_{12}, 51 wt.%	6.7 wt.%	93.3 wt.%	NA
**200°C**	LiC_{18}, 100 wt.%		3.1 wt.%	96.9 wt.%	**3.72 wt.%**
**240°C**	LiC_{18}, 30 wt.%	Graphite 2H, 70 wt.%	0.93 wt.%	99.07 wt.%	**2.1 wt.%**
**260°C**	Graphite 2H, 100 wt.%		0	100 wt.%	**0.88 wt.%**

Supplementary Note 1. Calculation of anode element weight ratio and leached lithium weight ratio in Supplementary Table 1

Anode elemental weight ratio: The anode elemental weight ratio was calculated based on the HEXRD results at different temperatures. For example, at 25°C, LiC_6 and LiC_{12} are at the phase weight ratio 49% and 51%, thus the total lithium weight ratio is (assuming 100g materials): [49×7/79+51×7/151]/100= 6.7 wt.%, while the carbon elemental weight ratio is: 1-6.7 wt.%= 93.3 wt.%. The same method is applied to the condition at 200°C, 240°C and 260°C.

Leached lithium weight ratio: From 25°C to 200°C, the weight ratio of leached lithium is calculated based on the same amount total materials. For example, assuming 100g materials at 25°C, then should be 6.7g lithium intercalated in 93.3g graphite; at 200°C, with 93.3g graphite, the intercalated lithium is 93.3g/96.9 wt.%×3.1 wt.%= 2.98g, thus the leached lithium is 6.7g - 2.98g=3.72g, so the leached lithium weight ratio at 200°C is 3.72g/100g=3.72 wt.%. Similarly, at 240°C, the intercalated lithium in 93.3g graphite is 93.3g/99.07wt.%×0.93wt.%=0.88g, thus the leached lithium weight is 2.98g-0.88g=2.10g (corresponding to a leached lithium weight ratio of 2.1% from 200°C to 240°C). Then at 260°C, the leached lithium is 0.88g, corresponding to a leached lithium weight ratio of 0.88% from 240°C to 260°C.

Supplementary Note 2. Calculation of the lithium consumption by SEI, PVDF and oxygen

At around 200°C, all the SEI have been decomposed. Thus, the amount of lithium consumed by SEI is the total leached lithium at 200 °C (i.e. 3.72 wt.% in Supplementary Table 1), which corresponds to 55.5 wt.% of total lithium (3.72%/6.7%).

To calculate the amount of lithium consumption by PVDF, assuming 0.2 g PVDF with 9.5 g graphite as the anode. Based on the Supplementary equation (1), to consume 0.2 g PVDF, the amount of lithium needed is: 0.2 g × (3×7 g/mol)/(128 g/mol) = 0.0328 g, which is 0.0328 g/9.5 g = 0.35 wt.% of lithium in the graphite anode. Thus, 0.35 wt.% of lithium in the anode is needed to consume all the PVDF.

$$3Li + (\text{-}CF_2\text{-}CH_2\text{-})_2 = \frac{1}{2}H_2(g) + 3LiF + (\text{-}CH{=}CH\text{-}CH{=}CH\text{-}) \quad \text{Supplementary equation (1)}$$

Compared with 6.7 wt.% of lithium in the lithiated graphite anode after charging (indicated by the Rietveld refinement of the anode after having been charged, as shown in Figure 2c and Supplementary Table 1), the amount of leached lithium consumed by PVDF is only 0.35/6.7 = 5.2 wt.% of the total lithium.

Therefore, by taking into the consideration of both SEI and PVDF lithium consumption, which should be 55.5 wt.% (SEI) + 5.2 wt.% (PVDF) = 60.7 wt.%. Therefore, before 200°C, 60.7 wt.% of total lithium can be consumed, while 39.3 wt.% of the residual liquid lithium can exist on the graphite surface at high temperatures, leading to the major potential hazards.

The consumption of lithium by oxygen occurred at different temperature were calculated based on the Supplementary equation (2):

$$4Li + O_2 = 2Li_2O \qquad \Delta H = -1197.46\ kJ/mol_{oxygen}\ [10] \quad \text{Supplementary equation (2)}$$

The entropy change of the above reaction based on the 1 mole oxygen is -1197.46 kJ/mol. In our oxygen exposure reaction, the amount of lithium is limited. Thus, based on lithium, the

reaction entropy is -299.4 kJ/mol_{Li} (-1197.46/4), which is also -299.4/6.95 =-43.14 kJ/g_{Li}. Therefore, the theoretical value of heat generation by inducing oxygen at different temperature can be calculated by multiply -43.14 kJ/g_{Li} and the amount of leached lithium at that temperature.

Supplementary Method

Materials. The dimensions of the electric vehicle-scale lithium-ion battery pouch cell are 227×160×7 mm. The cathode active material is $LiNi_{1/3}Mn_{1/3}Co_{1/3}O_2$; the anode composition is graphite (95wt.%), PVDF (2wt.%), and carbon black (3wt.%), with polyethylene (PE) as the separator. The battery cycle performance, charge/discharge curve, and battery specifications are listed below in Supplementary Figure 1. The capacity/voltage curves during charge/discharge between 2.5 and 4.2V @ 1C are shown in Supplementary Figure 1a. The pouch cell showed no capacity fading within 60 cycles under 1C charge/discharge, indicating a reasonable performance for electric vehicle usage. The battery was charged to 4.2 V under constant current (1/3C)-constant voltage (4.2 V, 1/20C cut-off current) after being charged for 2 cycles, then the pouch cell was carefully disassembled inside a glovebox. The lithiated graphite electrode was rinsed with Dimethyl carbonate (DMC) solvent followed by drying under vacuum overnight. The harvested lithiated graphite electrode showed typical gold color; see Supplementary Figure 1b. Lastly, the lithiated graphite powder was carefully scratched from the electrode and prepared for characterizations.

In situ high-energy XRD during heating with mass spectrometry. A beamline standard time-resolved high-energy X-ray diffraction (HEXRD) system[1] with a residual gas analyzer was utilized at Beamline 17 BM of Advanced Photon Source (APS)[2] to characterize the phase transformation during heating, with the X-ray wavelength of 0.24105Å. The lithiated anode powder (~2 mg) was loaded into a quartz capillary tube (7 mm) with a controllable heating unit and helium protective gas flow (Supplementary Figure 2). Ultrahigh-purity helium was used as the carrier gas (flow at 5 ml/min), and a residual gas analyzer (Pfeiffer Vacuum PrismaPlus® QMG 220) was utilized to measure the outgassing of the anode during heating. The heating rate was 2°C/min from room

temperature to 280°C; the HEXRD data acquisition rate was 30 seconds per image by the 2D detector. The 2D HEXRD data were then converted and analyzed with GSAS II software.

The *in situ* heating HEXRD and mass spectrometry (MS) technique is a standard characterization setup developed by the beamline scientist of Sector 17-BM in the APS of Argonne National Laboratory,[2] with the advantages of *in situ* HEXRD in monitoring the lattice evolution and MS to trace the gas release. It was previously used to monitor the phase transformations and gas evolutions of delithiated cathodes.[3,4]

For the *in situ* heating experiments on the lithiated graphite with the presence of electrolytes, the experiment setups are the same as dry lithiated graphite: about 2 mg lithiated graphite was loaded in the quartz capillary tube, then an additional 1 μL 1.2 M $LiPF_6$ in EC:EMC (3:7 volume) electrolyte was added, ethylene carbonate (EC), ethyl methyl carbonate (EMC). The only difference was that to preserve the electrolytes during the heating process, the helium gas flow was stopped during this measurement. Therefore, there were no MS gas signals for the experiment with the electrolyte.

DSC test with oxygen induction. The differential scanning calorimetry (DSC) tests were carried out using a DSC 214 Polyma (NETZSCH, Germany). The DSC tests were conducted at the ramping rate of 10°C/min in a nitrogen atmosphere with the gas flow rate set as 40 $mL \cdot min^{-1}$, then at the designed temperatures such as 200°C, 240°C, and 260°C; the DSC flow gas was swift to pure oxygen at the same rate. The heat generation was based on the weight of the lithiated anode, with typically 3 mg being loaded in the autoclave.

SEM and post-analysis. Scanning electron microscopy (SEM) characterizations were conducted to exam the morphological change of the lithiated graphite anode after the heating-induced lithium leaching. For Figure 4a in the main text, the material was collected from the graphite anode

laminate before charge. And for Figure 4c in the main text, the material was collected after the *in situ* HEXRD measurement with natural cooling to room temperature after reached 280°C.

In situ pair distribution function measurement and analysis. Synchrotron X-ray total scattering data were collected on beamline 11-ID-C at the Advanced Photo Source (APS), ANL. The rapid-acquisition PDF method was used with a wavelength of λ = 0.1173 Å.[5] A PerkinElmer amorphous Si two-dimensional image-plate detector (2048 × 2048 pixels and 200 × 200 m pixel size) was used at a distance of ~400 mm. The two-dimensional data were converted to one-dimensional XRD data using the GSAS-II software.[6] PDF data were obtained from Fourier transformation of the background and Compton scattering corrected data S(Q) in xPDFsuite software over a Q range of 0.4-19 Å-1.[7, 8] A Linkam THM600 furnace with temperature control <0.1°C was used to heat the lithiated powder, which was sealed in Kapton tape, see the setup as Supplementary Figure 15a. The heating rate was set at 5°C/min, then the sample was hold at each target temperature for at least 10 min before each PDF data acquisition. The PDF acquisition time was 20 min per image, and for each image a CeO_2 standard powder (CeO_2 NIST 647b) calibration was carried out. The furnace was calibrated between 30°C to 350°C by using a MgO lattice parameter as standard[9] before the experiment, see Supplementary Figure 15b.

Supplementary References

1. Chupas, P. J.; Chapman, K. W.; Kurtz, C.; Hanson, J. C.; Lee, P. L.; Grey, C. P., A versatile sample-environment cell for non-ambient X-ray scattering experiments. *J. Appl. Crystallogr.* **2008,** *41* (4), 822-824.
2. 17 BM, Advanced Photo Source, Argonne National Laboratory. (https://www.aps.anl.gov/Beamlines/Directory/Details?beamline_id=88)
3. Bak, S. M.; Nam, K. W.; Chang, W.; Yu, X. Q.; Hu, E. Y.; Hwang, S.; Stach, E. A.; Kim, K. B.; Chung, K. Y.; Yang, X. Q., Correlating Structural Changes and Gas Evolution during the Thermal Decomposition of Charged $Li_xNi_{0.8}Co_{0.15}Al_{0.05}O_2$ Cathode Materials, *Chem. Mater.*, **2013**, *25*, 337-351.
4. Hu, E. Y.; Bak, S. M.; Liu, J.; Yu, X. Q.; Zhou, Y. N.; Ehrlich, S. N.; Yang, X. Q.; Nam, K. W., Oxygen-Release-Related Thermal Stability and Decomposition Pathways of $Li_xNi_{0.5}Mn_{1.5}O_4$ Cathode Materials, *Chem. Mater.*, **2014**, *26*, 1108-1118.
5. Chupas, P. J.; Qiu, X.; Hanson, J. C.; Lee, P. L.; Grey, C. P.; Billinge, S. J. Rapid-acquisition pair distribution function (RA-PDF) analysis. *J. Appl. Crystallogr.* **2003**, 36 (6), 1342-1347.
6. Toby, B. H.; Von Dreele, R. B. GSAS-II: the genesis of a modern open-source all purpose crystallography software package. *J. Appl. Crystallogr.* **2013**, 46 (2), 544-549.
7. Yang, X.; Juhas, P.; Farrow, C. L.; Billinge, S. J. xPDFsuite: an end-to-end software solution for high throughput pair distribution function transformation, *visualization and analysis. arXiv preprint arXiv*:1402.3163 2014.
8. Adams, B. D.; Zheng, J.; Ren, X.; Xu, W.; Zhang, J.-G. Accurate Determination of Coulombic Efficiency for Lithium Metal Anodes and Lithium Metal Batteries. *Adv. Energy Mater.* **2018**, 8 (7), 1702097.
9. G. Fiquet, P. Richet and G. Montagnac, High-temperature thermal expansion of lime, preiclase, corundum and spinel, *Phys. Chem. Miner.,* **1999**, *27*, 103-111.
10. Chen, R. S.; Nolan, A. M.; Lu, J. Z.; Wang, J. Y.; Yu, X. Q.; Mo, Y. F.; Chen, L. Q.; Huang, X. J.; Li, H., The Thermal Stability of Lithium Solid Electrolytes with Metallic Lithium. *Joule* **2020,** *4* (4), 812-821.
